# Small Cell Variant of Intravascular Large B-Cell Lymphoma: Highlighting a Potentially Fatal and Easily Missed Diagnosis

**DOI:** 10.1155/2018/9413015

**Published:** 2018-04-03

**Authors:** Mahboubeh Rahmani, Stephanie Halene, Mina L. Xu

**Affiliations:** ^1^Department of Pathology, Harvard Medical School, Boston, MA, USA; ^2^Department of Internal Medicine, Hematology Division, Yale University School of Medicine, New Haven, CT, USA; ^3^Department of Pathology and Laboratory Medicine, Yale University School of Medicine, New Haven, CT, USA

## Abstract

**Context:**

Intravascular large B-cell lymphoma (IVLBCL) is a rare non-Hodgkin B-cell lymphoma with a poor prognosis. While typically described as comprising large atypical cells restricted to the lumina of small blood vessels, it can show variability in cell size.

**Objective:**

To report the clinicopathologic features of the IVLBCL with small cell morphology and discuss the practical implications of our findings.

**Design:**

We searched our archives for all IVLBCL diagnosed in our institution for the last 25 years (1992–2017). Slides were reviewed independently by two hematopathologists.

**Results:**

We found a total of 11 cases of IVLBCL. Bone marrow, brain, lymph node, pericardium, small bowel, and fallopian tube and ovary were the organs in which the lymphoma was initially diagnosed. One of the cases initially diagnosed in the marrow showed intrasinusoidal involvement by a small cell lymphoma; the diagnosis was confirmed by random skin biopsies showing intravascular large cells with the same phenotype. Retrospective review of the liver on this case also showed the intrasinusoidal involvement by the disease consisting of small cells. In another case, IVLBCL that was initially diagnosed in a small bowel biopsy was retrospectively found in a breast biopsy, but with small cell morphology.

**Conclusions:**

Our findings suggest that, in the presence of high clinical suspicion, IVLBCL should be high in the differential diagnosis when lymphoma is predominantly intravascular, even when the tumor cells are small. A timely diagnosis of this entity can be critical. Hence, awareness of a small cell variant of IVLBCL should be increased.

## 1. Introduction

Intravascular large B-cell lymphoma (IVLBCL) is a rare and usually aggressive extranodal mature non-Hodgkin B-cell lymphoma that is characterized by malignant lymphoid cells restricted to the lumen of blood vessels particularly capillaries. It usually presents in elderly adults with two major clinical presentations including a Western form in which symptoms are related to the main organ involved, predominantly with neurological or cutaneous involvement, and an Asian variant with multiorgan failure, hepatosplenomegaly, pancytopenia, and hemophagocytosis. B symptoms are common in both variants. A third isolated cutaneous variant is also described, which has a better prognosis in comparison to the first two variants as it is mostly confined to the skin [1].

Most of the patients have systemic disease at the time of diagnosis, and, although nonspecific, mental status change is the main presentation. However, since the tumor cells are predominantly circulating in small vessels without forming a mass, the diagnosis becomes challenging. Although cutaneous lesions including patches or plaques may not be the dominant presenting feature, when a high index of clinical suspicion is present, random multiple skin biopsies may be necessary to establish the diagnosis. It has been described that IVLBCL tends to colonize benign vascular neoplasms such as hemangiomas the biopsy of which may yield a higher positive rate of diagnosis in comparison to random skin biopsies [1, 2].

IVLBCL is invariably described as an angiotropic lymphoma composed of large-sized cells; however, in the current study, we searched our archives for all IVLBCL diagnosed in the Department of Pathology for the last 25 years (1992–2017) and found two cases of IVLBCLs, histopathologically presenting as small cell intravascular infiltrates with morphologic heterogeneity that appeared to be site-dependent. This unusual feature can be a major pitfall in obtaining a timely diagnosis in the setting of a rapidly progressive disease.

## 2. Materials and Methods

We retrospectively searched our archives for all IVLBCLs diagnosed in the Department of Pathology in the last 25 years (1992–2017), using a natural language search through the electronic database. Patients demographics and clinical information including the organs involved and clinical outcomes were studied; and all the pathology slides were reviewed looking for involvement by IVLBCL with small cell morphology. All H&Es and immunohistochemical studies of these cases were reviewed independently by two hematopathologists.

## 3. Results

Upon review of our archives in the department of pathology, we found 11 cases with the diagnosis of IVLBCL, six of whom were deceased and one had no clinical information/note in the system. The demographics, organs involved, and clinical outcomes are summarized in Table 1. Four of the cases had biopsy-proven CNS involvement. One of these presented with mental status change and another patient presented with worsening seizure activity and cognitive impairment. One of the deceased cases was diagnosed at autopsy with involvement of most organs. Three (27%) of the cases were diagnosed on a bone marrow biopsy specimen and three (27%) of the cases were diagnosed on a brain biopsy specimen. Lymph node (one case, 9%), pericardium (one case, 9%), small bowel (one case, 9%), and fallopian tube and ovary (one case, 9%) were the other organs yielding the initial diagnosis.

18% of our cases (2 of 11) showed intravascular cells of small size at the original biopsy site with establishment of the final diagnosis of IVLBCL at a second site. Both diagnoses were confirmed by the presence of large atypical cells in additional biopsies (Cases 1 and 2, see below for details).

### 3.1. Case 1: Clinical Presentation and Laboratory Findings

The patient was a 70-year-old man with history of hypertension and hyperlipidemia who presented with gait instability (2 weeks), chronic dry cough (4 months), weight loss (15-lb in 2 weeks), and fever (in 2016). His dry cough started 4 months prior to his presentation for which he underwent extensive work-up with no specific pathologic findings. He was prescribed different medications including prednisone for presumed COPD with no improvement.

Review of systems was significant for exertional dyspnea. Splenomegaly was noted on physical exam with no palpable lymphadenopathy. At the time of presentation, he had full strength (5/5) in upper and lower extremities with mild atrophy of proximal muscles. His gait was broad-based without difficulty turning.

Laboratory findings included prolonged PT and PTT along with mildly decreased factor VII level attributed to mild vitamin K deficiency or underlying liver disease; although transaminitis was noted, hepatic evaluation was negative for autoimmune and viral hepatitis. Chest CT-scan showed subsegmental left lower lobe acute pulmonary embolus (PE). Serum protein electrophoresis (SPEP) showed a discrete abnormal band, measuring < 0.2 g/dL identified to be IgM kappa by serum immunofluorescence studies (IFE). Electromyogram (EMG) and Nerve Conduction Studies (NCS) showed demyelinating axonal neuropathy concerning Guillain-Barre syndrome (GBS). A paraneoplastic autoantibody panel was negative. Serum-free light chains were normal.

### 3.2. Case 1: Hospital Course and Pathology Findings

During his hospitalization, the patient developed worsening weakness. Given concern for Guillain-Barre syndrome, treatment with plasma exchange (PLEX) was initiated (as IVIg was contraindicated due to PE) with no significant improvement.

**Figure 1:**
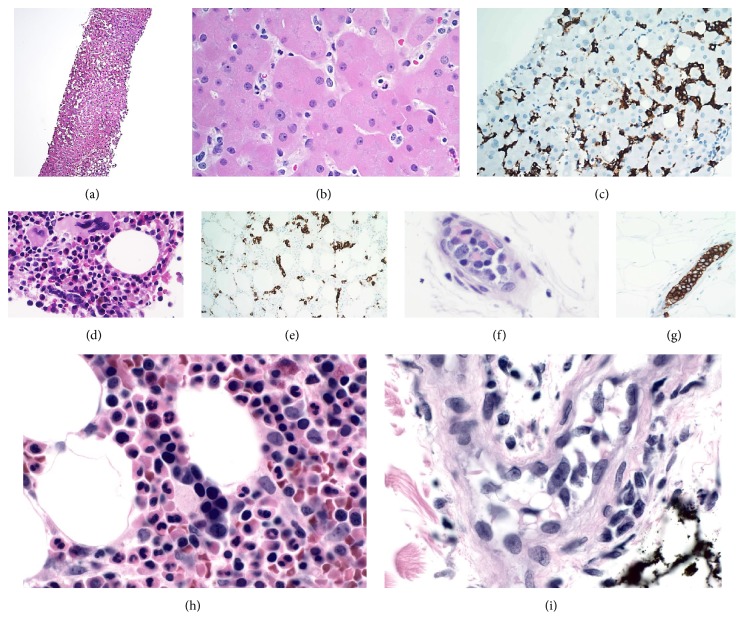
(a) Liver biopsy showing mild steatosis and dilated sinusoids, H&E stain, 4x magnification. (b) Extensive intravascular involvement of the liver by small atypical cells, H&E stain, 40x magnification. (c) Small intrasinusoidal lymphocytes highlighted by CD20, immunohistochemistry stain, 20x magnification. (d) The marrow showed involvement by atypical cells mostly of small size lymphocytes with an intrasinusoidal pattern, H&E stain, 40x magnification. (e) Small intrasinusoidal lymphocytes highlighted by CD20, immunohistochemistry stain, 20x magnification. (f, g) Subcutaneous vessel showing intravascular large atypical cells highlighted by CD20 confirming the diagnosis of IVLBCL, H&E, and immunohistochemistry stains, 40x magnification. (h, i) Small intrasinusoidal lymphocytes in the marrow in comparison with intravascular large atypical cells in the skin, H&E stains, 60x magnification.

Liver enzymes continued to rise with an increasing INR not responding to vitamin K supplementation. Consequently, a liver biopsy was performed, which was consistent with mild steatosis and dilated sinusoids (Figure 1(a)).

The patient had a prolonged hospitalization with multiple consultations involved with no unifying diagnosis. Due to a concern for an underlying malignancy, PET scan was performed which showed a hypermetabolic and enlarged spleen with patchy bone marrow hypermetabolism.

Due to the constellation of fever, splenomegaly, and increased bone marrow uptake on PET scan, he underwent a BM biopsy because of a concern for an underlying lymphoma. Flow cytometry of the marrow showed involvement of marrow with monoclonal B-cell LPD comprising about 5% of total cellularity. The immunophenotype was CD19+ CD20+ CD5dim+ CD23− CD10− CD103− CD38dim+ CD11c− FMC7dim+ CD43− IgG− IgMdim/− and kappa light chain restricted. Ki-67 proliferation index was attempted but was very difficult to evaluate given the focality of the lesion. It was estimated at approximately 40–50% within the abnormal lymphocyte population.

Karyotype and FISH results for the IGH, BCL6, MYC, 15q, 17p (TP53), 13q, CDKN2C, and CKS1B loci in bone marrow cells were normal and a PCR test for cyclin D1 mRNA level was not elevated. The histology of the marrow showed involvement by non-Hodgkin B-cell lymphoma with a striking intrasinusoidal pattern. However, the infiltrative lymphocytes were predominantly small in size, which raised the possibility of a splenic marginal zone lymphoma as well as splenic diffuse red pulp small B-cell lymphoma. However, given the patient's clinical presentation and his hospital course, IVLBCL was considered by pathology to be more likely despite the lack of large cell proliferation. After consulting with oncologists as well as the primary medicine team, random skin biopsies were performed, which showed morphologically classic IVLBL (Figures 1(d)–1(i)).

On retrospect, review of the liver biopsy showed extensive intravascular involvement of the liver by small atypical cells highlighted by CD20 (Figures 1(b) and 1(c)). The immunophenotype was the same for both small and large lymphoma cells. At the time of this report, the patient has 6 cycles of R-CHOP and experienced significant improvement in the movements of bilateral lower extremities. He achieved complete remission and has just received dose modified BEAM with autologous stem cell rescue.

### 3.3. Case 2: Clinical Presentation

The patient was a 31-year-old woman who presented with severe headache, dizziness, and facial edema in the beginning of her pregnancy (in 1997). Initially, it was attributed to her pregnancy, but due to persistence of the symptoms, she underwent an extensive work-up including lumbar puncture and MRI with no specific pathologic findings. She was started on corticosteroids with some improvement.

At 16-week gestational age, she developed abdominal pain followed by a spontaneous abortion; however, the pain was not relieved by pain medications. Further work-up revealed superior mesenteric vein thrombosis and subsequently the patient underwent resection of the infarcted bowel.

### 3.4. Case 2: Pathology Findings and Patient Follow Up

**Figure 2:**
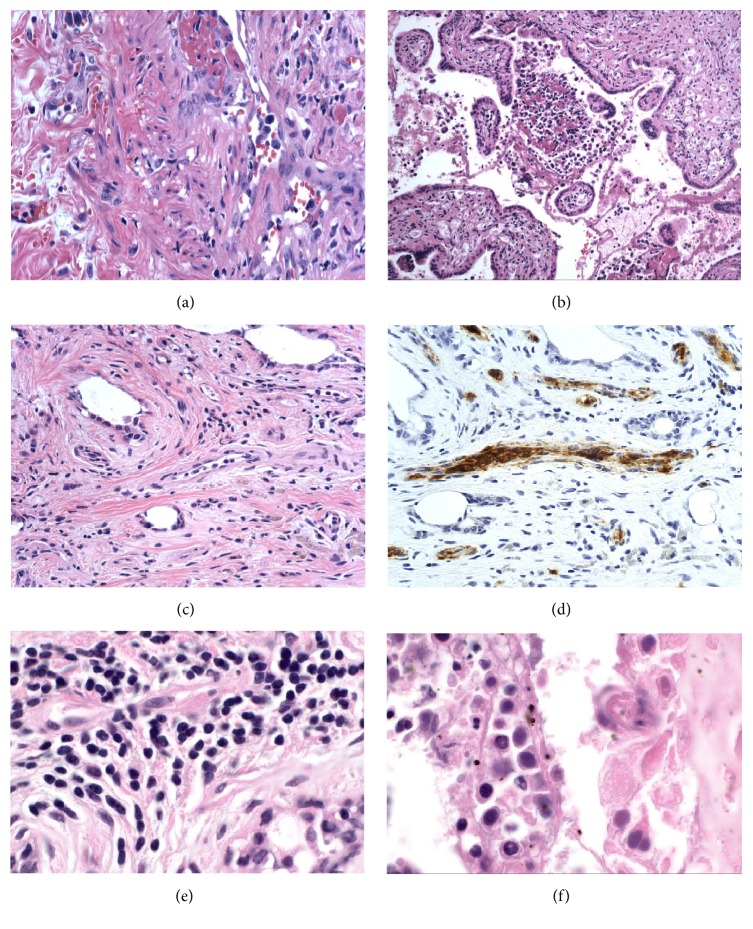
(a) Histopathologic exam of the small bowel revealed the diagnosis of IVLBCL with intravascular large atypical cells, H&E stain, 20x magnification. (b) Placenta also showed involvement of the disc (maternal blood) by IVLBCL including large atypical cells, H&E stain, 20x magnification. (c, d). Small atypical cells were seen in the small blood vessels of the breast, highlighted by CD20, H&E, and immunohistochemistry stains, 40x magnification. (e, f) Small lymphoma cells involving breast in comparison with large cells involving placenta, H&E stains, 60x magnification.

Histopathologic exam of the small bowel revealed the diagnosis of IVLBCL (Figure 2(a)). The mesenteric vasculature was extensively involved with an occlusive vasculopathy. In the midst of occluded vessels were large, malignant cells with a high nuclear:cytoplasmic ratio and highly irregular nuclear outlines. The vascular lesion affected predominantly medium-to-large-sized mesenteric veins, although numerous smaller vascular channels contained scattered malignant cells. The lymphoma cells were CD45+ CD20+ CD10− CD5− EBV− (by EBER).

Retrospective review of the placenta taken after the spontaneous abortion showed involvement of the disc (maternal blood) by IVLBCL including large atypical cells (Figure 2(b)). Subsequently, bone marrow biopsy was performed and showed involvement of the marrow by lymphoma. The patient received three cycles of CHOP and matched related donor allogeneic stem cell transplant (HSCT). At the time of submission of this article, the patient is alive and in clinical remission.

Upon the review of patient's previous biopsies, we encountered a breast biopsy that had been given a benign diagnosis, performed one month prior to the diagnosis of IVLBCL, was made. As it is shown in Figure 2(c), small atypical cells are present in the small blood vessels of the breast, highlighted by CD20 (Figure 2(d)). The immunophenotype was the same as large cells seen in small bowel. This serves as a striking example of IVLBCL presenting with small cell morphology in restricted sites.

## 4. Discussion

Intravascular large B-cell lymphoma is an aggressive disease with a poor prognosis. Previous studies have shown that although any organ may be involved, neurological signs and symptoms are the most common presentation of the disease [3–8]. Neuropathic deficits are very heterogeneous, including mental status changes, transient ischemic attack, paraparesis of the extremities, and defect in motor functions [9, 10]. In the current study, central nervous system, bone marrow, pericardium, liver, skin, small bowel, and fallopian tube and ovary were organs with biopsy-proven involvement by lymphoma.

To the best of our knowledge, there is no report of placental involvement with a confirmed diagnosis of intravascular large B-cell lymphoma. Hanaoka and Mio et al. reported a case of a 33-year-old pregnant lady presented with hemophagocytic syndrome. Histologic examination of placenta revealed involvement by B-cell lymphoma with diffuse infiltration of large, atypical lymphoid cells involving the intervilli spaces, similar to our report (case 2). While this case was not subclassified as intravascular large B-cell lymphoma, the images demonstrated some features that are suggestive of such involvement.

Although IVLBCL is described as involvement of small vasculature by large neoplastic cells, variability in these characteristics has been reported in rare cases, including cells of small sizes, extravasation of neoplastic cells, and involvement of larger veins and arteries [11]. However, this phenomenon is not mentioned in most textbooks or described as a caveat in WHO diagnostic criteria.

In this small review, 18% of our cases (2 of 11) showed intravascular cells of small size followed by a definitive diagnosis of IVLBCL based on additional biopsies. While the diagnoses were eventually rendered based on the presence of large neoplastic cells in other sites, this necessity for large-cell morphology may cause undue delay in the management of a rapidly progressive disease.

Bone marrow involvement by intrasinusoidal B-cell lymphoma raises the differential diagnosis of splenic diffuse red pulp small B-cell lymphoma and splenic marginal zone lymphoma; however, IVLBCL should also be in the differential as it could morphologically present with small size cells. This diagnostic dilemma is treacherous given that IVLBCL is clinically aggressive while the other two are indolent lymphomas. A high clinical suspicion should warrant timely further investigation. Additional markers, such as for B-cell precursor phenotype (TdT, CD34, and CD99) can help rule out an acute lymphoblastic leukemia. Proliferation index can be difficult to evaluate, as we discovered in our case, in the histological context of focal infiltration.

Currently, diagnostic certainty requires the finding of large cells. It is possible that, with better molecular profiling in the future, ancillary tests can assist the diagnostician.

It remains mechanistically unknown why the tumor is confined to intravascular spaces. Expression of the chemokine receptor CXCR3 on tumor lymphocytes and its ligand, CXCL9, on endothelial cells is proposed by Kato et al. as a potential mechanism; however, it is a single case study [12]. Ponzoni et al. have shown a model of central nervous system (CNS) invasion by malignant lymphoma, requiring ICAM and integrin family proteins for tumor cells to first dock and then lock with endothelial cells. Degradation of the extracellular matrix with the aid of matrix metalloproteinases helps the invasion of the brain parenchyma. Based on this model, it is proposed that the absence of adhesion molecules such as beta1-integrin (CD29) or ICAM-1 (CD54) in the malignant lymphocytes or matrix metalloproteinases (including MMP-2 or MMP-9) are implicated in restricted intravascular pattern of IVLBCL [13, 14]. Saurel et al. have shown marked expression of angiogenic factors, including VEGFA, VEGFC, FIGF, and SPP1 by qRT-PCR in a case of cutaneous IVLBCL compared to other types of DLBCLs which may explain the apparent tropism of IVLBCL to vascular neoplasms including benign hemangiomas [15]. More studies are needed to better clarify the exact mechanism. Elucidation of the intravascular tropism may shed light on why certain large tumor cells are trapped in particular organ sites while smaller-sized tumor cells are found in others.

Our findings suggest that, in the presence of high clinical suspicion, including rapid clinical deterioration and multiorgan failure, IVLBCL should be in the differential diagnosis even when the intravascular tumor cells are small in size. A timely diagnosis of this entity can be critical. Hence, we recommend promoting awareness of a small cell variant of IVLBCL; however, without simultaneous occurrence of classic large cell IVLBCL in additional anatomical sites, the recognition of this small cell variant could be very subjective and thus prone to diagnostic error.

## Figures and Tables

**Table 1:** Patient's demographics and clinical information.

Case #	Age at the time of diagnosis/gender	IVLBCL first diagnosis year	IVLBCL first diagnosis organ/size of the cells	Other organs' involvement/size of the cells	Clinical information
1	70 y/M	2016	Bone marrow, small	Liver, small Skin, large	Alive, on chemo

2	31 y/F	1997	Small bowel, large	Breast, small Placenta, large BM, large	Alive, 18 years following matched related donor allogenic stem cell transplant

3	62 Y/M	2017	Lymph node, large	N/A	Alive, on chemo

4	75 y/M	2016	Brain, large	N/A	Alive, on chemo

5	80 y/M	2015	BM, large	N/A	Deceased

6	74 y/F	2011	Brain, large	N/A	Deceased

7	60 y/M	2011	Brain, large	N/A	Deceased

8	63 y/F	2010	BM, large	N/A	No note in the system

9	76 y/F	2003	Tube and ovary, large	N/A	Deceased

10	47 y/F	1996	Pericardium, large	N/A	Deceased

11	35 y/F	1993	Autopsy	Multiple organs involved, large	Deceased
